# Opioid Analgesic Prescription in French Children: A National Population-Based Study

**DOI:** 10.3390/ijerph182413316

**Published:** 2021-12-17

**Authors:** Samira Choufi, Simon Mounier, Etienne Merlin, Emmanuelle Rochette, Jessica Delorme, Nicolas Authier, Chouki Chenaf

**Affiliations:** 1 Pédiatrie, CHU Clermont-Ferrand, F-63000 Clermont-Ferrand, France; samirac90@hotmail.fr (S.C.); smounier1@chu-clermontferrand.fr (S.M.); e_merlin@chu-clermontferrand.fr (E.M.); 2 CIC 1405, Unité CRECHE, INSERM, Université Clermont Auvergne, F-63000 Clermont-Ferrand, France; 3 CHU Clermont-Ferrand, Inserm 1107, Neuro-Dol, Service de Pharmacologie Médicale, Centres Addictovigilance et Pharmacovigilance, Centre Evaluation et Traitement de la Douleur, Université Clermont Auvergne, F-63003 Clermont-Ferrand, France; jdelorme6@gmail.com (J.D.); nauthier@chu-clermontferrand.fr (N.A.); cchenaf@chu-clermontferrand.fr (C.C.); 4 Observatoire Français des Médicaments Antalgiques (OFMA)/French Monitoring Centre for Analgesic Drugs, Université Clermont Auvergne-CHU Clermont-Ferrand, F-63001 Clermont-Ferrand, France; 5 Institut Analgesia, Faculté de Médecine, F-63001 Clermont-Ferrand, France

**Keywords:** analgesics, pediatrics, codeine, morphine, tramadol, pain

## Abstract

Codeine use was restricted in 2013 and is currently contraindicated for children below the age of 12 years. We examined how the prescription of opioid analgesics in children in France evolved between 2012 and 2018. Our population-based study from the SNIIRAM database (National System of Health Insurance Inter-Regime Information) was designed to determine trends in opioid prescription from 2012 to 2018 in all French children. The number of children who received at least one opioid prescription gradually declined from 452,665 in 2012 (347.5 children per 10,000) to 169,338 in 2018 (130.3 children per 10,000). This decrease was especially marked for codeine (36 children per 10,000 in 2018 vs. 308.5 children per 10,000 in 2012), whereas the number of tramadol prescriptions increased by 171% in 2018 (94.6 children per 10,000). Despite the increase, strong opioids still formed only a small proportion of prescriptions (2.6 children per 10,000 given opioids in 2018). Overall opioid prescriptions in French children dramatically decreased between 2012 and 2018, probably owing to restrictions on the use of codeine. Codeine has been partly replaced by tramadol. Morphine is still probably underused. This suggests that opioids are being used less often for pain management in children.

## 1. Introduction

Opioid analgesics have been used for many years for the treatment of pain in both adults and children. In the last decades, an increase in the prescriptions of opioid analgesics has been observed, in both adults [1] and children [2,3]. Some studies using national data have analyzed trends in prescribing opioid analgesics in children [2,4], but such studies are scant. In addition, new measures have brought restrictions on the use of certain opioids. This is so for codeine, a prodrug whose main active metabolite is morphine. Owing to serious adverse effects in children, the EMA (European Medicines Agency) in 2012, [5] the FDA in the United States in 2013 [6], and the ANSM in France in April 2013 [7] recommend restricting the use of codeine in children under the age of 12 years and after tonsillectomy and/or adenoidectomy. The use of tramadol has also become controversial in recent years, leading to the same restrictions as for codeine in the United States since 2017 [8]. In France, it remains available from age 3, subject to increased vigilance in its use [9].

The use of opioid analgesics therefore continues to be a subject of debate, with safety still an issue. The last few years have seen changes in prescriptions as safety flaws have been highlighted. Pediatric opioid epidemiology requires more data to determine the status of opioid analgesic consumption in children.

Based on a collection of exhaustive national data, our objective was to examine how the dispensations of opioid analgesics evolved in children in France between 2013 and 2018.

## 2. Materials and Methods

### 2.1. Participants

In this population-based study, all children aged 15 and under between 2012 and 2018 and registered in the SNIIRAM (National System of Health Insurance Inter-Regime Information) were included. The age limit of 15 years was chosen because the marketing authorization for all opioid analgesics in France after age 15 years is the same as for adults.

### 2.2. Study Design

This study was a repeated cross-sectional analysis. We determined the number of children who had received at least one oral analgesic opioid prescription between 1 January 2012 and 31 December 2018. The prescribed analgesic opioids were dispensed by private pharmacies or hospitals prescription and included: buprenorphine, codeine, dihydrocodeine, fentanyl, hydromorphone, morphine, opium, oxycodone, and tramadol. For the analysis, we classified individual opioid type as weak (codeine, dihydrocodeine, opium, and tramadol) or strong (buprenorphine, fentanyl, hydromorphone, morphine, oxycodone). We further classified them, according to the World Health Organization (WHO) definitions, into Step 2 analgesics (tramadol, codeine, opium, and dihydrocodeine) for moderate to severe pain and/or in case of failure of step 1 (non-opioid) analgesics (paracetamol and ibuprofen) for mild pain; and Step 3 analgesics (morphine, oxycodone, hydromorphone, buprenorphine, and fentanyl) for severe pain and/or in case of failure of Step 2 analgesics.

### 2.3. Data

Data concerning opioid prescriptions were collected from the SNIIRAM database covering 98.8% of the French population (over 66 million people) [10]. This database contains (i) demographic data: sex, date of birth, and information on complementary private insurance coverage indicating low-income status, (ii) presence of chronic disease, represented by a list of 30 long-term diseases (Affection Longue Durée [ALD]) with their associated International Classification of Diseases (ICD–10) codes, and (iii) medications, recorded as dispensed preparation packs including ATC (Anatomical Therapeutic Classification) codes, dosage form, nature of prescriber, date of dispensing, and name of dispensing pharmacy (anonymized). All reimbursements of analgesic opioids were identified by their ATC codes (morphine N02AA01 and N02AA51, oxycodone N02AA05 and N02AA55, fentanyl N02AB03, buprenorphine painkillers N02AE01, hydromorphone N02AA03, tramadol N02AX02 and N02AX52, codeine N02AA59 and N02AA79, dihydrocodeine N02AA08 and N02AA58, opium N02AA02). We accessed all these data from 2012 to 2018. Data were only from prescriptions dispensed in pharmacies. Only the oral forms were included; the IV forms being for hospital use were excluded.

### 2.4. Analyses

We determined the number of children who received at least one prescription for an opioid analgesic between 01 January 2012 and 31 December 2018. The analysis was carried out semester by semester, by age range ([0–2], [3–5], [6–11], [12–15] years), type of opioid, type of prescriber, and medical specialty (general medicine, pediatrics, or surgical). We also calculated the prevalence of children who received at least one prescription for an opioid analgesic in each semester between 2012 and 2018 and annually for years 2012 and 2018. The denominator used to calculate the prevalence was the number of children aged 15 and under. Comparisons of analgesic opioid prescription were made with the Cochrane-Armitage trend test applied to test the difference from 2012 to 2018. All statistical analyses were performed using SAS^®^ software version 9.4 (SAS Institute, Cary, NC, USA).

### 2.5. Ethics

This study was approved by the French data protection authority (Commission nationale de l’informatique et des libertés CNIL, reference DE-2016-110).

## 3. Results

**Figure 1:**
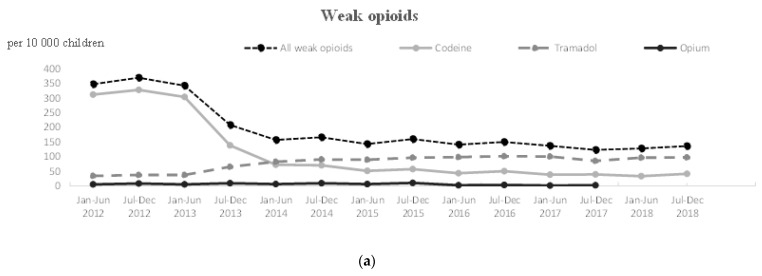

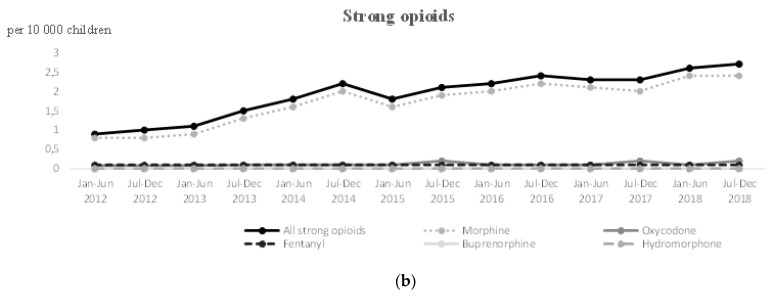
Trends of opioids use in children aged from 0 to 15 years between 2012 and 2018. (**a**) For weak opioids; (**b**) for strong opioids.

**Figure 2:**
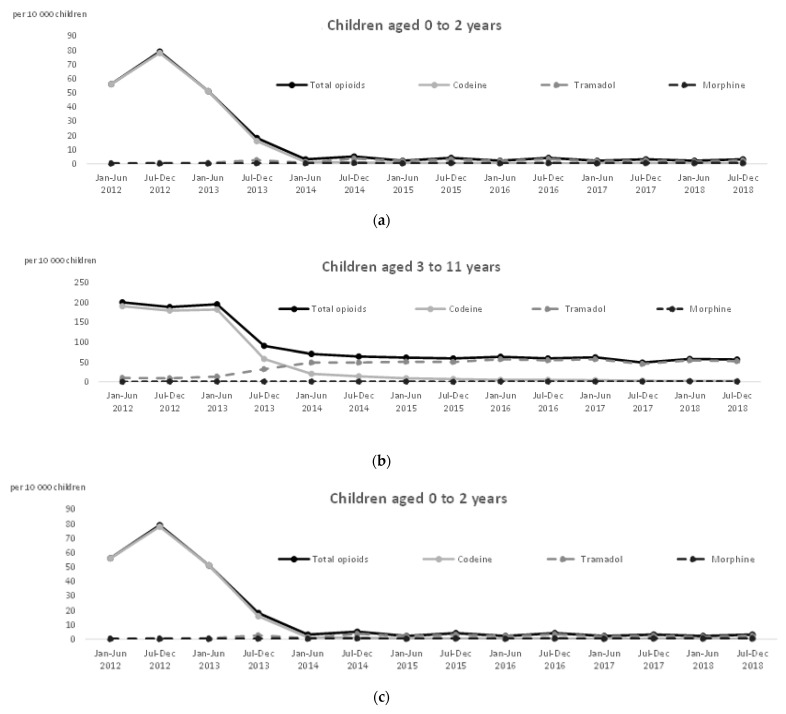
Evolution by age group of dispensations of opioid analgesics between 2012 and 2018. (**a**) For children aged 0 to 2 years; (**b**) for children aged 3 to 11 years; (**c**) for children aged 12 to 15 years.

Table 1, Table 2 and Table 3 compare characteristics of patients who used opioids between 2012 and 2018. Figure 1 and Figure 2 show the semester-by-semester change in the number of children who received at least one opioid analgesic between 2012 and 2018.

In 2012, 452,665 children in France received at least one opioid prescription (347.5 children per 10,000) (Table 1). Of these children, 449,068 or 99% received at least one weak opioid, and 1226 or 0.2 % received at least one strong opioid (mostly morphine; 0.70 children per 10,000) (Table 1). Codeine was the most often prescribed opioid (308.5 children per 10,000), far ahead of tramadol (34.7 children per 10,000) (Table 1).

The number of children who received at least one opioid prescription gradually declined to 169,338 in 2018 (−62.5%, 130.3 children per 10,000) (Table 1). This decrease concerns in particular codeine (36 children per 10,000 in 2018, i.e., a drop of 88.2% compared to 2012) (Figure 1**a**). Even so, 3.1 per 10,000 children received off-label codeine in 2018 (Table 1).

Conversely, the number of children who received at least one tramadol prescription increased by 171% in 2018 (94.6 children per 10,000) (Figure 1a). The same trend was observed for strong opioids (+173%, prevalence of 0.9 children per 10,000 in 2012 vs. 2.6 in 2018. However, strong opioids represented a small proportion of prescriptions (Figure 1b).

The number of children with chronic disease who were given an opioid decreased from 9 per 10,000 in 2012 to 3 per 10,000 in 2018 (Table 1).

The number of children who received opioids decreased in all age groups (−95.8% among 0–2-year-olds (Figure 2a), −79.3% among 3–11-year-olds (Figure 2b), and −23.7% among 12–15-years-olds (Figure 2c)).

In children aged 3–11 years, tramadol was the only oral step 2 analgesic that had marketing authorization. Nevertheless, its increase did not offset the decrease in codeine (190 per 10,000 children for codeine in January–June 2012 vs. 54 per 10,000 for tramadol in January–June 2018) (Figure 2b).

The number of children who received morphine also increased in all age groups between the first 6 months of 2012 and the last 6 months of 2018 (+1035% among 0–2-year-olds; +173.4% among 3–11-year-olds; +86.8% among 12–15-year-olds) (Table 2). However, the absolute number of prescriptions remained low. In the 0–2-year-old age group, we observed more off-label deliveries of tramadol than of morphine, which can be delivered from 6 months in oral form (Table 3).

## 4. Discussion

In France in 2012, 347 per 10,000 children received at least one delivery of an opioid analgesic. During the same year in the United States, 2.3 million children aged 0–17 out of 69.9 million (or 290 per 10,000 children) received at least one prescription for an opioid analgesic [11]. Similar studies carried out in Australia, Norway, Sweden, and Denmark found lower prescription prevalence than in France in 2012 [12]. As in France, codeine was the most often prescribed opioid in all these countries except Denmark, where tramadol predominated. Other studies have shown higher prevalence of outpatient treatment prescriptions in France, and in particular analgesic therapy in children [13,14]. This can be explained by different reimbursement systems among countries [15].

We observed a significant drop in the dispensing of opioid analgesics between 2012 and 2018, probably due to restrictions in the use of codeine in April 2013 in France. A study that evaluated the impact of codeine restrictions found an 84% drop in prescriptions in France between 2010 and 2015, 44% in Germany, and 33% in the United Kingdom [16]. We observed the same phenomenon with a drop of 88.2% in codeine dispensation from 2012 to 2018. Despite these trends, we observed that 4143 children under the age of 12 received at least one dispensation of codeine in 2018 but with no data on the reason for the prescription. In 2017 in Australia, codeine accounted for 44.5% of all prescriptions and 10.5% for patients below 12 years [4]. The main indication reported was cough. In previous studies, otitis media and gingivostomatitis were the most challenging painful conditions in children to be managed without codeine, along with postoperative pain, especially post-tonsillectomy [17,18]. In the example that involved gingivostomatitis, previous codeine prescriptions may be explained by the absence of other oral Step 2 analgesics. Indeed, for this predominant pathology in children below 3 years of age, only codeine was available, at least in France (tramadol was available for children 3 years old and above) [17].

Despite an increase in prescriptions of tramadol, these only partially replaced those of codeine. With tramadol authorized to be marketed for children aged 3 years and above in France, it was considered a possible alternative to codeine in the report of the High Authority for Health in 2016 (HAS) [9]. Nevertheless the HAS recommended caution in the use of tramadol because it can also have severe effects; because it acts as a prodrug for strong opioids, its metabolism follows the same pathway as codeine [19]. Although tramadol is thought to be slightly less addictive than codeine, interactions with other agents may result in serotonin syndrome. Orliaguet et al. reported the case of an ultra-fast metaboliser child aged 5 years hospitalised for a coma after taking tramadol following adenotonsillectomy surgery for obstructive sleep apnea [20]. Several cases of intentional and unintentional intoxication have also been described [21]. In April 2017, the Food and Drug Administration (FDA) restricted the use of tramadol in all children younger than 12 years, and after tonsillectomy and/or adeneidectomy in children between 12 and 18 years. The FDA has also warned against the use of tramadol in obese adolescents between the ages of 12 and 18 and children with conditions such as obstructive sleep apnea or severe lung disease. [8]. The issue of acute intoxication is probably not so imperative in the setting of chronic pain, as side effects depend on genetics, dose, and on the intensity of the pain. Hence close attention must be paid mostly to the initial administrations, especially in very young children.

It was to be expected that oral morphine formulations would be increasingly prescribed after codeine was no longer authorised in very young children. Although the relative increase was considerable (>1000% in 0–2-year-old children), the absolute number of prescriptions remained well below that of tramadol in the same age group. Although available in France from age 6 months in oral formulation, morphine seems to be underused, maybe due to persistent misconceptions about its use in children. Indeed, the use of morphine may be perceived by parents and health professionals as having more risks associated with side effects and misuse [22,23,24]. However, there is no evidence that morphine has more side effects than other opioids [25]. Some authors have even suggested that it replace all codeine prescriptions.

The prescribing trends for weak opioids in France appear to be similar to trends in other countries, but this is not true for strong opioids. The other strong opioids were very seldom prescribed. Although prescribed off-label, oxycodone represented approximately two thirds of opioid prescriptions in 2017 in Australia [4] and in 2014 in the United States was the most often prescribed oral opioid for children [12]. In 2015, the FDA authorized prolonged-release oxycodone for children aged 11–16 years, “with pain severe enough to require daily, around-the-clock, long-term opioid treatment for which alternative treatment options are inadequate” [25]. Yet in France, oxycodone is prescribed only for adults. According to the 2016 HAS report on alternatives to codeine, oxycodone could be an option in situations where the other drugs fail, are contraindicated, or lead to serious adverse events (SAEs). The report nevertheless specifies that pediatric use requires additional investigation [9]. Indeed, oxycodone has the same metabolic pathways as codeine and tramadol [26,27]. In two studies comparing the effectiveness of oxycodone to ibuprofen in orthopedic pain, oxycodone was not more effective than ibuprofen and may even have more side effects [28,29]. The risk of addiction must also be considered. After the creation of a veritable “opioid overflow” in the United States, numerous cases of misuse have been observed in adolescents in the years following the marketing of oxycodone [30,31]. Large-scale studies therefore seem necessary before considering the use of oxycodone in children.

The overall decrease in opioid dispensations we observed suggests either that analgesic drug prescriptions are no longer adequate in children, especially in the context of acute moderate-to-severe pain [32] in very young children, or else that previous prescriptions were largely unnecessary. A study carried out in a pediatric emergency department reports that 64.6% of children aged 6–24 months did not receive opioid analgesics vs. 47.6% for older children for the same reasons (fractures, burns) [33]. Younger children thus seem less likely to receive opioid analgesics. In another study, among 93% of participating physicians who had answered “yes” to the question, “Do you think that the use of opioids is safe in children?”, only 43% used them routinely and 30% in therapeutic doses [34]. Concerns raised included fear of side effects, dependence, or tolerance; the lack of usual prescriptions of opioids in pediatrics; and the lack of knowledge and training on the use of opioids in children [35].

Conversely, the decline in the dispensing of opioid analgesics can be viewed positively if the intent is to use them more appropriately and relevantly. The issue of the use of opioid analgesics is part of the desire not to subtreat pain in children and to prevent their undesirable effects and misuse, particularly by adolescents and younger people [36]. It seems difficult to reason about their use without considering the reason for the prescription and the acute or chronic nature of the pain. The most current guidelines for the use of opioid analgesics tend to adapt the choice of analgesic to the etiology of the pain, considering studies that compared the effectiveness of treatments. The HAS report recommends first-line non-steroidal anti-inflammatory drugs (NSAIDs), particularly ibuprofen, for moderate to severe pain [9]. In fact, the non-inferiority or even the superiority of NSAIDs has been demonstrated in certain situations, particularly in trauma [37,38]. Opioids should be considered from the outset in events of severe pain or in events of failure of paracetamol and ibuprofen. Recent studies and guidelines on post-operative analgesia consider pain management without opioids as much as possible in many situations [39,40]. Consensus and interdisciplinary collaborations seem necessary for the relevance and safe and appropriate use of opioids in children.

Our study and discussion were limited in that they did not clearly relate prescriptions to indications. In addition, there was no guarantee that a reimbursement of an opioid prescription for a child corresponded to its actual use by that child. We could also not determine whether this decrease in the prescription of opioid analgesics meant previous overuse or current underuse for pain in children. Likewise, we had no data concerning non-opioid analgesics (paracetamol, NSAIDs), which could offer an alternative to codeine. A recent study observed a significant increase in paracetamol prescriptions in France between 2006 and 2015 [41]. However, that study did not target only the pediatric population.

## 5. Conclusions

In conclusion, our study shows that overall opioid prescriptions in French children dramatically decreased after codeine was contraindicated. Codeine has been partly replaced by tramadol, and morphine may be underused. These findings suggest that opioids are being used less for pain in children.

## Figures and Tables

**Table 1:** Characteristics of children who received at least one opioid: comparison between 2012 and 2018.

	2012 (*n*)	per 10,000	2018 (*n*)	per 10,000	*p*-Value	Variation between 2012 and 2018 (%)
**Children who received at least one opioid dispensation**	452,665	347.5	169,338	130.2	<0.0001	−62.5
**Mean age (** **±sd)**	7.3 ± 4.8		10.7 ± 4.2			
**Age distribution**						
[0–2]	85,469	65.6	3554	2.7	<0.0001	−95.9
[3–5]	126,348	97.0	26,092	20.0		−79.4
[6–11]	118,714	91.1	46,614	35.8		−60.7
[12–15]	122,134	93.7	93,078	71.6		−23.6
Sex						
Female	209,903	161.1	85,585	65.8	<0.0001	−59.2
Male	242,762	186.4	83,753	64.4		−65.5
**Weak opioids**	449,068	344.8	166,773	128.2	<0.0001	−62.8
Codeine	401,872	308.5	47,295	36.3	<0.0001	−88.2
Dihydrocodéine	47	0.0	39	0.0	0.0002	−17
***Codeine off-label prescription***	-	-	4143	3.1		
Tramadol	45,313	34.7	123,046	94.6	<0.0001	+171.5
***Tramadol off-label prescription***	649		2417	1.8		+272
Opium	9058	6.9	-	-	-	
**Strong opioids**	1226	0.9	3359	2.6	<0.0001	+173.9
Morphine	1041	0.7	3032	2.3	<0.0001	+191.2
Fentanyl	157	0.1	188	0.1	<0.0001	+19.7
Buprenorphine	9	0.0	2	0.0	0.50	+77.7
Hydromorphone	2	0.0	5	0.0	0.009	+150
Oxycodone	112	0.0	279	0.2	<0.0001	+149.1
**Mode of exercice**						
Private practice	257,734	197.9	80,236	61.7	<0.0001	−68.8
Hospital	170,035	130.5	75,648	58.1	<0.0001	−55.5
**Speciality**						
General Medicine	313,827	240.9	95,464	73.4	<0.0001	−69.5
Pediatrics	45,938	35.2	10,601	8.1	<0.0001	−76.9
Surgical specialties	44,080	33.8	25,147	19.3	<0.0001	−42.9
Chronic illness	11,909	9.1	4455	3.4	<0.01	−62.5

Note: The presented values are for the total number of children aged 15 or below in France (i.e., 13,023,428 in 2012 and 12,999,208 in 2018) (source: INSEE or National Institute of Statistics and Economic Studies). The sum for the different classes of opioids may not correspond to the total, because the figures represent the number of children who have received at least one opioid prescription. (For example, based on a single prescription, a patient could have received two weak opioids or a weak and a strong opioid.).

**Table 2:** Morphine dispensations by age group, comparison of year 2012 and year 2018.

	January–June 2012		July–December 2012		January–June 2018		July–December 2018		Variation (%) *
	*n*	per 10,000	*n*	per 10,000	*n*	per 10,000	*n*	per 10,000	
0 to 2 years	40	0.06	48	0.07	320	0.5	454	0.7	+1035
3 to 11 years	256	0.4	278	0.4	793	1.2	700	1.0	+173.4
12 to 15 years	351	0.5	372	0.5	632	0.9	656	1.0	+86.8

* Variation (%) between January–June 2012 and July–December 2018.

**Table 3:** Number of children aged 0 to 2 years who received an opioid, comparison of year 2012 and year 2018.

	January–June 2012		July–December 2012		January–June 2018		July–December 2018		Variation (%) *
	*n*	per 10,000	*n*	per 10,000	*n*	per 10,000	*n*	per 10,000	
Total Opioids	37,074	56.9	51,723	79.4	1455	2.2	2107	3.2	−94.3
Codeine	36,707	56.3	51,301	78.7	170	0.2	194	0.3	−99.4
Tramadol	288	0.4	369	0.5	968	1.4	1464	2.2	+408.3
Morphine	40	0.06	48	0.07	320	0.5	454	0.7	+1035

* Variation (%) between January–June 2012 and July–December 2018.

## Data Availability

It is currently not possible to share study datasets. The data from the SNIIRAM (National System of Health Insurance Inter-Regime Information) database were the subject of a specific extraction request. The permission for sharing or open data needs to obtain new approval by the French data protection authority (Commission nationale de l’informatique et des libertés CNIL).
